# Lactoferrin Levels in Cerebrospinal Fluid Exhibit Differential Associations with Alzheimer’s Disease

**DOI:** 10.21203/rs.3.rs-9678397/v1

**Published:** 2026-07-07

**Authors:** Feiyang Zhao, Raquel Puerta, Yaxi Wang, Eva Beckett, Pablo Garcia Gonzalez, Sergi Valero, Pilar Sanz, Tiffany F. Kautz, Jose E. Cavazos, Maria Victoria Fernandez, Amanda Cano, Sudha Seshadri, Merce Boada, Valentina R. Garbarino, Agustin Ruiz-Laza, The Global neurodegeneration Proteomics Consortium (GNPC)

**Affiliations:** a Glenn Biggs Institute for Alzheimer's & Neurodegenerative Diseases, The University of Texas at San Antonio, San Antonio, TX, USA; b Department of Microbiology, Immunology and Molecular Genetics. Long School of Medicine, The University of Texas at San Antonio, San Antonio, TX, USA; c Ace Alzheimer Center Barcelona-Universitat Internacional de Catalunya, Barcelona, Spain; d Department of Biochemistry and Structural Biology. Long School of Medicine. University of Texas San Antonio, TX, USA; e CIBERNED, Network Center for Biomedical Research in Neurodegenerative Diseases, National Institute of Health Carlos III, Madrid, Spain; f South Texas Medical Science Training Program, University of Texas Health San Antonio, San Antonio, TX, USA; g School of Public Health, Boston University and the National Heart, Lung, and Blood Institute Framingham Heart Study, Boston, MA, USA; h Department of Neurology, Boston University School of Medicine, Boston, MA, USA; i Department of Cell Systems & Anatomy, The University of Texas at San Antonio, San Antonio, TX, USA

**Keywords:** Alzheimer’s disease, Lactoferrin, CSF, Plasma, Biomarkers

## Abstract

Lactoferrin has been proposed as a minimally invasive biomarker for Alzheimer’s disease (AD), but its relationship with established AD pathology remains uncertain. We analyzed paired cerebrospinal fluid (CSF) and plasma SOMAscan proteomic profiles from 1,367 participants in the ACE Alzheimer Center Barcelona cohort, focusing on two lactoferrin-targeting SOMAmers, Seq.14755.4 (LTF1) and Seq.2780.35 (LTF2). LTF1 and LTF2 showed distinct distributions and weak concordance across fluids, indicating that they should not be treated as interchangeable lactoferrin measures. In CSF, LTF2 was associated with lower Aβ42 and AD biomarker-defined status, whereas LTF1 showed subgroup-dependent associations with tau markers that were partly influenced by major CSF proteomic axes and reference-gene adjustment. Neither CSF nor plasma LTF signals predicted conversion from mild cognitive impairment to dementia. GNPC analyses supported the non-interchangeability and compartment specificity of LTF signals and identified a reproducible plasma LTF2-centered network enriched for innate immune and granule biology. These findings suggest that lactoferrin-related signals are assay-, compartment-, and network-context dependent rather than standalone AD biomarkers.

## Introduction

Alzheimer's disease (AD) is the most common cause of dementia in the elderly, which is characterized by progressive cognitive decline, loss of neurons, amyloid-beta (Aβ) deposition, and tau pathology [1–2]. Combinations of clinical assessment, structural/functional neuroimaging, and evaluation of cerebrospinal fluid (CSF) based pathological markers, are considered well-established AD biomarkers with validated diagnostic precision to detect AD progression [2–3]. Despite recent advancements in diagnostic tools, these CSF and imaging-based markers remain invasive and/or expensive, highlighting an urgent need for new diagnostic biomarkers to address these limitations. Furthermore, the underlying cause of neurodegenerative disease remains unclear. Even the widely accepted hypothesis, that Aβ aggregation leads to a pathological cascade, cannot alone fully explain the pathological initiation and progression of AD [3–4]. In the last decades, there has been growing recognition that immune system declines during aging and persistent inflammation induced by sterile and microbial inflammatory responses contribute to Aβ deposition, progressive synaptic dysfunction, neuronal loss, and ultimately AD [5]. In support of this hypothesis, infections of the central nervous system (CNS) are known to be associated with AD-like pathology [6–10]. Furthermore, oral and gut microorganisms are now known to play a role in the cascade of immune dysregulating events preceding AD [11–12]. The involvement of pathogen-targeting agents and viral infection–related markers in the brain such as amyloid aggregation, reinforces the potential association between AD and immunologic response of the CNS to infection(s) [12–13]. These pieces of evidence suggest an important role of both local and systemic inflammation towards AD pathogenesis.

As primary effector molecules of innate immunity, antimicrobial proteins and peptides may contribute significantly to AD development [14–15], specifically Aβ pathology [16–19]. Lactoferrin (LTF), an iron-binding glycoprotein involved in innate immunity, acting as part of the body’s first line of defense against bacterial, viral, fungal, inflammatory and carcinogenic compounds [20], has attracted attention in recent years for its potential role in neurodegeneration [21–23]. Beyond exocrine secretion by epithelial cells in most mucosal tissues, it is also expressed by the cells of the innate immune system throughout the body, including peripheral neutrophil leukocytes and monocytes, and microglia within the brain [24–25]. In neutrophils, LTF is stored in the secondary granules and released into the blood or infected tissues at high concentrations during inflammation. LTF’s expression and release occurs not only in peripheral tissues but also in the CNS, where microglial cells can release LTF through degranulation, particularly under inflammatory conditions [25–26].

In the last decade, several studies have reported altered LTF abundance in biological fluids of AD patients. For instance, through a cross-sectional investigation, Carro, et al. found that decreased salivary LTF expression can discriminate AD patients from healthy controls more accurately than Aβ42 and total-tau (t-tau) in CSF [27]. The same research group revealed that decreased salivary LTF levels can reliably distinguish prodromal AD and clinical AD groups from frontotemporal dementia (FTD) with high accuracy [28]. Through a later study, they also demonstrated negative associations between salivary LTF and Aβ load, middle temporal cortex thickness, increased FDG uptake in posterior cingulate cortex, and poorer memory [29]. Studies from this research group have consistently validated their previous findings, that measures of salivary LTF may be a promising non-invasive and cost-effective AD biomarker. However, a subsequent, independent follow-up study in a mixed memory clinic cohort did not find an association between LTF levels in CSF or saliva and cognitive diagnoses or pathological AD markers (t-tau, p-tau, Aβ) [30]. These conflicting findings from different research groups highlight the need for standardization across sample collection, processing and assessment methods, and suggest that LTF may have a complex, context-dependent relationship with specific neurodegenerative disease pathology and progression—relationships which are not fully captured or detectable by current studies and evaluation assays and methods. Disagreement between these studies may stem from several complex aspects of neurodegenerative disease pathology and the biology of LTF that is endogenously generated by a variety of cell types including: immune cell, glial cells, epithelial cells [25, 31]. LTF not only exists in the neural system but also within the immune, digestive, circulatory and reproductive systems, so it is important to consider that circulating LTF levels are potentially modulated by multiple tissues, organ systems, environmental/dietary exposures and potentially even some genetic factors.

LTF measurements also illustrate a broader issue relevant to high-throughput affinity-based proteomics: when multiple binders target the same protein, their signals may not be interchangeable because they can differentially reflect isoforms, post-translational states, epitope accessibility, or protein–complex context. Accordingly, assay performance and interpretability should be supported by orthogonal evidence, including (i) platform-level technical evaluations of large-panel aptamer assays [32] and (ii) studies that incorporate independent direct quantification (e.g., mass spectrometry) alongside affinity-based readouts to contextualize and interpret protein-specific behavior [33] In the present work, we therefore treat the two LTF-targeting SOMAmer signals as distinct readouts rather than assuming equivalence.

To explore the potential involvement of LTF in AD and strengthen our understanding of the role of LTF in AD, we examined data from a novel and independent cohort. We utilized existing proteomic data from the ACE Alzheimer Center Barcelona (Spain), which includes a large number of participants with paired CSF and plasma proteomic profiles measured by the high-throughput SOMAscan 7K platform. By leveraging this well-characterized dataset combined with readily available detailed clinical phenotyping, we aimed to clarify the relationship between LTF and AD across CSF and plasma, and to assess whether LTF holds promise as a disease-associated or stage-specific biomarker in these two biofluids. This study provides an opportunity to re-evaluate the role of LTF in AD pathogenesis with improved statistical robustness and biological resolution. Finally, to address the need for independent validation beyond a single clinical setting, we additionally evaluated whether our key conclusions replicate within the harmonized, multi-cohort proteomics framework of the Global Neurodegeneration Proteomics Consortium (GNPC) [34].

## Results

### LTF abundance in CSF and Plasma

**Fig. 1.**
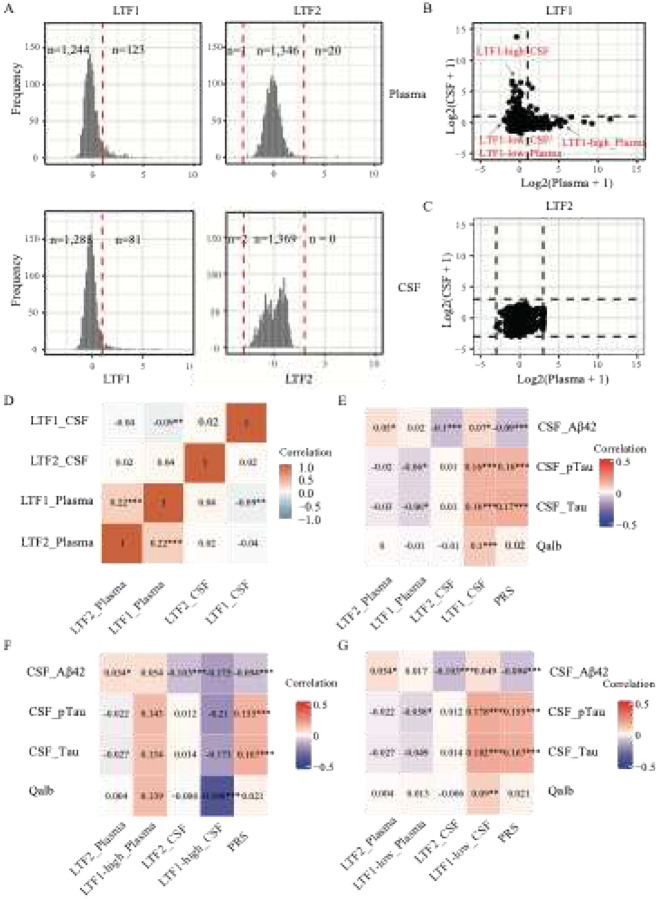
Distributions of SOMAmer signals targeting lactoferrin (LTF) and their associations with Alzheimer's disease (AD) biomarkers. **A**: Distributions of two SOMAmers targeting LTF (LTF1 on the left, LTF2 on the right) in plasma (top) and cerebrospinal fluid (CSF) (bottom). Red dotted lines indicate the cutoffs defined as mean + standard deviations for LTF1 and mean ± 3 standard deviations for LTF2. **B–C**: Comparisons of LTF1 and LTF2 signals between CSF and plasma; dotted lines represent the cutoffs shown in panel A. **D**: Heatmap showing the Spearman correlation between LTF1 and LTF2. False Discovery Rate (FDR) was used for multiple corrections of the p-values together. **E–G**: Heatmaps showing Spearman correlations between the two SOMAmers, polygenic risk scores (PRS), and AD biomarkers. Panel E displays results for all samples; panels F and G show correlations after stratifying LTF1 levels into high and low protein expressors for LTF1, respectively. *, p < 0.05; **, p < 0.01; ***, p < 0.001. FDR was used to perform multiple corrections of the p-values for LTF1/LTF2 in plasma and CSF separately.

To investigate whether LTF changes contribute to AD progression and the possibility to serve as a potential biofluid biomarker for AD, we accessed the plasma and CSF profiles of 1,369 individuals from the ACE CSF cohort. In this cohort, two samples lacked detectable signals for both LTF-targeting SOMAmers. Analysis included a total of 792 females and 577 males. The subjects consisted of 113 (8.27%) healthy controls; 833 (60.9%) diagnosed with MCI at baseline–of whom 44.1% converted to dementia during the follow-up visit, with CSF samples collected within 3 months of first visit–and 394 (28.2%) subjects diagnosed with dementia at the time of the lumbar puncture (Supplementary Table S2). The mean age at the time of lumbar puncture was 72.7 ± 8.8 years. As expected, the frequency of the *APOE* ε4 allele was higher in the dementia group (21.9%) compared to MCI group (19.9%) and control group (13.1%). Detailed demographic and clinical characteristics are summarized in Supplementary Table S2. We first investigated the distribution of all SOMAmers and noticed that distribution patterns of most SOMAmers did not follow a normal distribution including LTF1/2 in both CSF and plasma (Fig. 1A). For each individual, two SOMAmers that target LTF were measured in both CSF and plasma. For LTF1 (seq.14755.4) most individuals exhibited low expression in both CSF (132 ± 79) and plasma (370 ± 469), while a small subset showed high expression in one or the other fluid (Fig. 1A, B). The other SOMAmer (LTF2; seq.2780.35) displayed high expression in plasma (1,260 ± 4,703) for a small group of individuals (Fig. 1A, C) and another group of samples showed CSF-specific high expression (CSF: 19,951 ± 20,804). This result indicates that LTF1 and LTF2 possibly represent two different isoforms or different conformational/binding statuses of LTF. To further confirm this hypothesis, we compared LTF expression between the two SOMAmers. The correlation analysis reveals the signal between the two SOMAmers shows weak significant correlation only in plasma (Spearman’s ρ = 0.22; adjusted p-value < 0.001), while there was almost no significant correlation in CSF (Spearman’s ρ = 0.02; adjusted p-value > 0.05; Fig 1D). Meanwhile, results revealed no significant correlation between CSF and plasma (Spearman’s ρ = 0.02; adjusted p-value > 0.05) for LTF2, with a similarly negligible negative correlation for LTF1 (Spearman’s ρ = −0.09; adjusted p-value < 0.01; Fig 1D). To examine whether the distribution of LTF1 in CSF is associated with blood-brain barrier integrity, we performed Spearman correlation analysis between LTF1, LTF2 and the albumin quotient (Qalb; a measure of blood–brain barrier (BBB) leakage), revealing that only CSF LTF1 levels (Spearman’s ρ = 0.1; adjusted p-value < 0.001; Fig. 1E), not LTF2 (Spearman’s ρ = −0.01; adjusted p-value > 0.05), weakly associated with Qalb.

### LTF Levels and AD Biomarkers

We sought to determine whether LTF expression is associated with established CSF AT(N) biomarkers in the ACE CSF cohort. To address this, we performed Spearman correlation analysis between LTF1 and LTF2 signals with Aβ42, t-181 and p-tau181 (Fig 1E). We found that LTF1 in CSF showed a weak positive correlation with total-tau (Spearman’s ρ = 0.16; adjusted p-value < 0.001), p-tau181 (Spearman’s ρ = 0.16; adjusted p-value < 0.001) and a negligible correlation with Aβ42 (Spearman’s adjusted p-value = 0.07; adjusted p-value < 0.05). In contrast, LTF2 showed a weak negative correlation with Aβ42 (Spearman’s ρ = −0.10; adjusted p-value < 0.001) and no significant association with p-tau181 (Spearman’s ρ = 0.01; adjusted p-value > 0.05) or t-tau (Spearman’s ρ = 0.01; adjusted p-value > 0.05). Based on the distribution patterns of LTF1 and LTF2 in CSF and plasma (Fig. 1A) and preliminary inspection, we hypothesized that the weak correlation might reflect the heterogeneity in expression across subgroups of participants. Furthermore, the distribution of LTF1 has a long tail and displayed a non-normal distribution, suggesting it may be expressed in only a subset of individuals. LTF2 appears to follow a normal distribution, with a few distinct outliers. To test this hypothesis, we stratified the samples based on LTF1 signal intensity: high (≥ mean + SD) and low (< mean + SD) and remove the outliers for LTF2. We then repeated the correlation analysis. For LTF2, results remained unchanged (Fig 1F; Fig S1). In the LTF1-low group, the associations with AD biomarkers were stable (Fig 1G; Fig S1), however, for LTF1-high groups, the direction of the associations reversed, and correlation coefficient increased, particularly for Aβ (Spearman’s ρ = −0.175, adjusted p-value = 0.120), and p-tau181 (Spearman’s ρ = −0.21, adjusted p-value = 0.061, Fig 1F; Fig S1). Notably, these coefficients were slightly stronger than those observed between AD polygenic risk score (PRS) and AD biomarkers, although the increase in explained variance remained modest (~2.0% for p-tau181, 0.2% for t-tau, and 2.2% for Aβ42).. Our recent work showed that variability in CSF proteomes is dominated by a ventricular-volume–related dilution axis, implying that CSF biomarker signals can be confounded by physiological variability [35]. To investigate its impact on the LTF–AD-biomarker relationship, we first examined correlations of LTF1/LTF2 with the first two proteomic principal components (PC1–PC2) and with reference genes (GAGE2A, OPCML). The result reveals LTF1 correlated with PC1 in the low-expression group (Spearman’s ρ = −0.373, adjusted p-value < 0.001; Fig. S2) but not in the high-expression group (Spearman’s ρ = 0.009, adjusted p-value > 0.05; Fig. S2). LTF2 showed no association with PC1 (Spearman’s ρ = −0.004, adjusted p-value > 0.05; Fig. S2).

To assess the influence of potential confounders, we performed sensitivity analysis to re-estimated the associations between CSF LTF1/LTF2 and AD biomarkers using linear models that adjusted for sex, age, age^2^, proteomic principal components (PC1–PC2), and reference-gene abundance as sensitivity analysis (Supplementary Tables S3–S6). After adjusting for PC1, LTF1 was not associated with Aβ42 (β = −0.005; p = 0.089; Supplementary Table S3), but it remained negatively associated with p-tau (β = −0.167; p < 0.001; Supplementary Table S3). In stratified analyses, LTF1 continued to associate with p-tau in the high-expression group after PC1 adjustment (β = −0.144; p = 0.003), whereas the apparent positive association in the low-expression stratum (β = 0.383; p < 0.001) disappeared after additional adjustment for reference genes (β = 0.010; p = 0.837) and showed a negative association after adjustment for PC1 (β = −0.167; p = 0.001). For LTF2, the association with Aβ42 (β = −0.112; p < 0.001) was partially attenuated by reference-gene adjustment (β = −0.087; P < 0.001) but was unchanged by PC1 (β = −0.113; p < 0.001) (Supplementary Table S6). Collectively, these results suggest that the LTF1 signal in the low-expression group was largely biased by physiological (dilution) variation before adjustment, whereas variation in high-expression LTF1 and in LTF2 is more likely to be pathologically relevant.

**Fig. 2.**
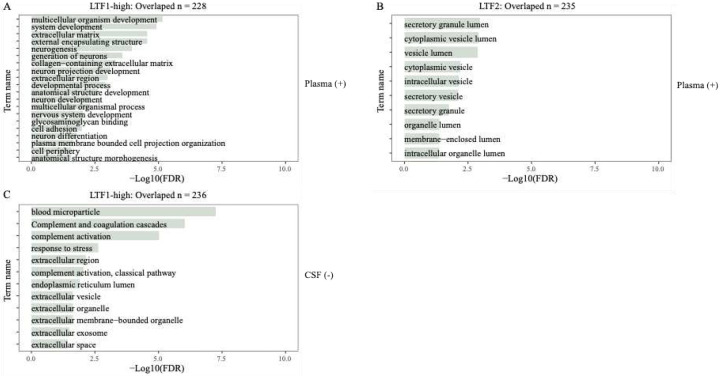
Gene Ontology (GO) enrichment analysis of lactoferrin (LTF)-associated proteins identified from SOMAscan plasma and cerebrospinal fluid (CSF) proteomic data. **A:** Significantly enriched GO biological process terms among proteins positively correlated with the plasma-derived LTF1 signal in individuals classified as high expressors (+ (n= 226)). **B:** GO cellular component terms enriched among proteins positively correlated with the plasma-derived LTF2 signal (n = 226). **C:** GO biological process and cellular component terms enriched among proteins negatively correlated with the CSF-derived LTF1 signal in the LTF1-positive group (n= 236). Enrichment significance is represented by −log10(FDR)-adjusted p-values.

**Fig. 3.**
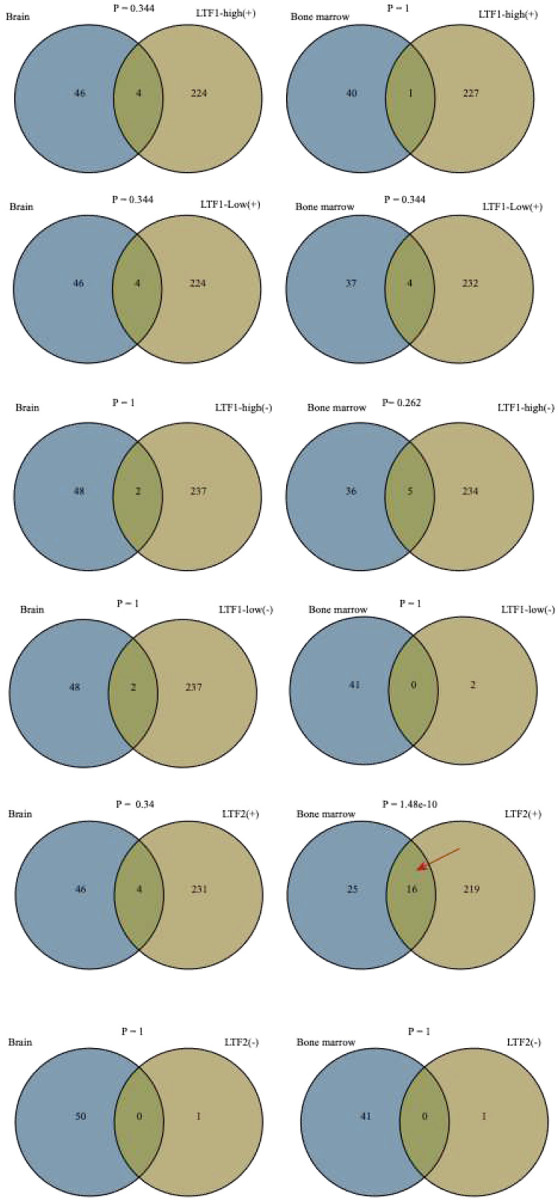
Overlap between lactoferrin (LTF)-derived correlation signatures in plasma and tissue-specific co-expression modules. Columns show tissue modules from the Human Protein Atlas database (Brain, #237 and Bone marrow, #154 blue circles). Rows stratify our signatures by (i) LTF1 expression stratum—LTF1-high vs. LTF1-low and (ii) correlation sign with the LTF signal: “+” = top positively correlated genes, “−” = top negatively correlated genes. For LTF2, correlations were computed across all samples (no expression stratification). In each panel, the right circle is the LTF-derived signature (yellow) and the left circle is the tissue co-expression set (blue). Statistical significance was assessed with Fisher’s exact test (background = all tested genes after QC, N= 5,419). False discovery rate (FDR) corrections were applied for LTF1 and LTF2 separately.

We then compared the LTF1 and LTF2 signal levels across participants with different AD pathological features. The results showed that LTF2 levels in CSF were significantly associated with AD status defined by AT(N) biomarker (p = 0.0051; Fig S3) and show trend toward an association with clinical symptom syndromic state (Kruskal-Wallis, p = 0.050; Fig S3), while LTF1 levels were not. The difference between AD and Control groups is statistically significant (adjusted p-value = 0.02). To determine the biological relevance of these SOMAmer signals, we performed pathway enrichment analysis on the top co-regulated proteins for LTF1 and LTF2. For LTF1 (in both plasma and CSF), enriched terms could not be found before stratification, however, in the LTF1-high group, we observed enrichment for neural-related pathways in the plasma analysis, including “neurogenesis”, “generation of neurons”, “neuron projection development”, and “neuron differentiation” (Fig 2A). Proteins correlated with LTF1 in CSF were enriched in immune-related categories such as “blood microparticle” and “complement and coagulation cascades” (Fig 2C). To further explore the differences of high and low populations, we analyzed the LTF-associated protein networks using SomaScan data. Correlation analyses between LTF1/LTF2 and all other quantified proteins were performed, and significantly coregulated proteins were compared with LTF interactors identified in single-cell RNA-seq data from brain and bone marrow. Before stratification, no enrichment overlap was found for LTF1 in CSF or plasma. However, in the positive group, which had limited statistical power, a nominally significant overlap with bone marrow–derived co-regulated genes was observed (p-value = 0.0328), although this association was not significant after multiple-testing correction, adjusted p-value = 0.262; Fig 3).

LTF2 showed modest enrichment for terms like “secretory granule lumen” and “vesicle lumen” (Fig. 2B). Overlap with bone marrow-derived networks was also confirmed (adjusted p-value = 1.48e-10). Together, these findings suggest that LTF1 and LTF2 are associated with distinct proteomic profiles and biological processes, possibly reflecting different aspects of the LTF locus biology and AD pathology. Importantly, the diagnostic relevance of LTF1 appears to be limited to specific subgroups of samples that have higher expression for LTF1 signal.

### CSF and Plasma LTF Levels Are Not Associated with AD Progression or Genetic Risk

**Fig. 4.**
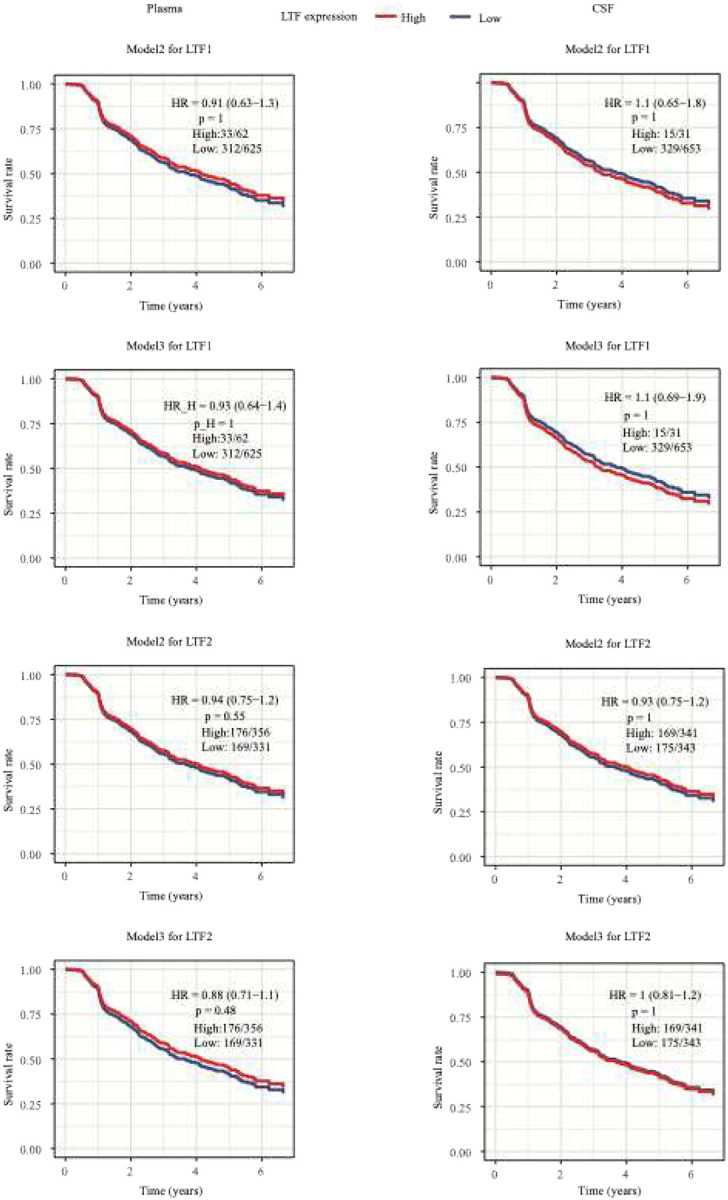
Survival analysis for lactoferrin (LTF) using nested models. The left column shows results based on plasma data; the right column shows results based on CSF data. For LTF1, survival rates were compared between LTF1 high and LTF1 low. For LTF2, samples were split by the median (High ≥ median; Low < median). Model1: Surv(Time(years), status) ~ LTF + Age_LP + sex + education + mmse_csf; Model2: Surv(Time(years), status) ~ LTF + csf_ p_tau + PRS + APOE + Age_LP + sex + education + mmse_csf. HR represents the hazard ratio of the positive group, 95% confidence intervals is included, and p is the corresponding p-value. In each panel, the conversion ratio is shown as conversion number / total follow-up participants.

We next examined whether LTF expression is associated with the progression from MCI to dementia. Using Cox regression models, we found that neither LTF1 nor LTF2 expression in CSF or plasma was significantly associated with disease progression. To account for potential confounding variables, we implemented nested Cox regression models. The second model included adjustments for age, sex, years of education, and MMSE score, while the third model further adjusted for PRS, p-tau181 levels, and APOE genotype. Across all models, LTF1 and LTF2 levels in both CSF and plasma were not significantly correlated with conversion from MCI to dementia (Fig. 4).

Additionally, we assessed whether variation in LTF expression could be driven by genetic risk for AD. However, no significant associations were found between AD-associated genetic variants and LTF expression levels in either CSF or plasma. These findings suggest that, despite the known roles of LTF in immune response and neuroinflammation, LTF expression levels in CSF and plasma do not appear to reflect AD progression or genetic susceptibility. This may indicate that LTF biomarker potential in CSF and plasma may be limited to identifying the presence of disease pathology, rather than providing a biomarker read for disease risk phenoconversion or genetic risk stratification in AD.

### GNPC validation replicates non-interchangeability, compartment specificity, and network-level structure

To address the need for validation of LTF measurements, we evaluated whether key structural conclusions are replicated in the harmonized GNPC multi-cohort framework [34]. Analyses included 5 CSF datasets (baseline n range: 117–1,370) and 20 EDTA plasma datasets (baseline n range: 95–3,150), with correlation and network analyses performed separately by fluid and cohort.

First, across GNPC datasets, LTF1 and LTF2 were only weakly and heterogeneously coupled, supporting the premise that the two binders should not be treated as interchangeable measures of a single biological signal. In CSF, cohort-level LTF1–LTF2 Spearman correlations were near zero (ρ range: −0.139 to 0.055; median ρ = −0.059 across estimable cohorts), and in EDTA plasma correlations were similarly modest (ρ range: −0.030 to 0.246; median ρ = 0.057), with some cohorts not estimable due to limited usable variance. Full cohort-level statistics are provided in Supplementary Tables 7 and 8.

Second, GNPC CSF analyses reproduced a consistent feature observed in our primary cohort: LTF1 tracked major proteomic axes of variation more strongly than LTF2. In CSF, LTF1 showed stronger correlations with Comp.1 and Comp.2 than LTF2 across key cohorts; for example, in a large CSF dataset (n = 1,156), LTF1 correlated with Comp.1 (ρ = 0.371; p = 5.27×10^−39^) and Comp.2 (ρ = 0.581; p = 2.84×10^−105^), whereas LTF2 showed minimal association with Comp.1 (ρ = 0.006; p = 0.850) and Comp.2 (ρ = 0.042; p = 0.156). These patterns support the interpretation that LTF1 is more tightly linked to broad proteomic structure in CSF, whereas LTF2 behaves more independently from the dominant proteomic components. These relationships are summarized in Supplementary Figure 4, with full cohort-level values provided in Supplementary Table 7.

Third, among GNPC cohorts with paired CSF–plasma sampling at the person level, within-individual cross-compartment concordance was essentially absent, reinforcing compartment specificity. For our paired cohort (Q cohort, N= 1,368), CSF vs. EDTA plasma correlations were near zero for both LTF1 (ρ = −0.022; p = 0.407) and LTF2 (ρ = 0.010; p = 0.701), and similarly near zero in other paired cohorts. These paired cross-compartment results are summarized in Supplementary Figure 5, with full cohort-level statistics provided in Supplementary Table 9.

Finally, cross-cohort network analysis provided the strongest validation signal: LTF2 yielded a substantially larger and more reproducible directed consensus network than LTF1 (Supplementary Figure 6), particularly in EDTA plasma. At an 80% directed consensus threshold (⩾16/20 plasma datasets; ⩾4/5 CSF datasets), EDTA plasma LTF2 produced a 40-marker positive consensus set and a 2-marker negative consensus set, whereas EDTA plasma LTF1 yielded only 4 positive and 1 negative consensus markers. The EDTA plasma LTF2 positive consensus included proteins with clear innate immune/granule biology (e.g., ELANE, MPO, LCN2, MMP9, BPI, S100A9, PRTN3, CAMP), and meta-analytic summary correlations for key consensus markers were robust (pooled |r| often ~0.33–0.48 across contributing datasets). In contrast, CSF consensus structure was comparatively limited (LTF1: 2 positive markers; LTF2: 6 positive markers at the same threshold), and overlap between CSF and plasma consensus sets was minimal, again, consistent with compartment-specific regulation. Functional enrichment of the EDTA plasma LTF2 positive consensus set converged on innate immune/neutrophil and secretory granule biology (Supplementary Figure 7 and Supplementary Table 13).

**Figure 5.**
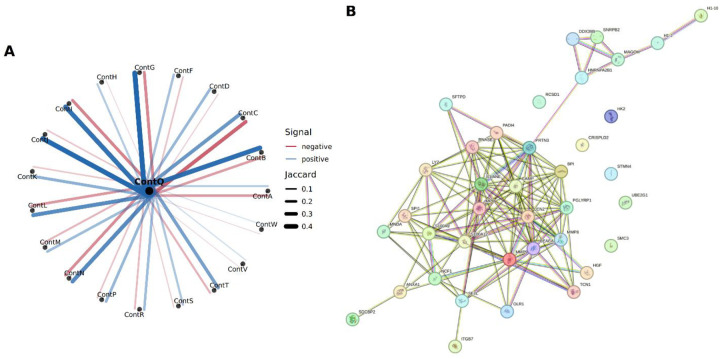
ACE-anchored recovery and STRING interaction structure of the plasma lactoferrin (LTF2) signal. (A) Cross-cohort recovery of the plasma LTF2-centered network using the ACE cohort within GNPC (ContQ) as the anchor dataset. (B) STRING interaction network of the plasma LTF2 positive consensus set, showing a strongly interconnected structure (38 mapped nodes, 137 edges, 14 expected edges, average node degree 7.21, clustering coefficient 0.609, PPI enrichment p < 1.0 × 10^−16^). These results support that the strongest GNPC-replicated signal reflects a shared and biologically coherent molecular program for LTF2.

Given that plasma LTF2 yielded the strongest GNPC validation signal, we next used the ACE cohort within GNPC (ContQ in the GNPC) as an explicit anchor to test whether the corresponding network biology was recoverable across fully independent studies. This analysis showed that the plasma LTF2 network identified in ACE was reproducibly recovered across multiple independent cohorts, particularly for the positive signal set, indicating that the main-study signal reflects shared cross-study biology rather than a cohort-specific pattern (Figure 5A). STRING analysis of the plasma LTF2 positive consensus set further showed that these proteins form a highly non-random interaction network comprising 38 mapped nodes and 137 edges, compared with 14 expected by chance, with an average node degree of 7.21, an average local clustering coefficient of 0.609, and a protein-protein interaction (PPI) enrichment p-value < 1.0 × 10^−16^ (Figure 5B). Together with the enrichment results shown in Supplementary Fig. S7 and Supplementary Table S13, these data indicate that the plasma LTF2 signal represents a biologically coherent and reproducibly shared molecular program centered on innate immune and granule biology.

## Discussion

Salivary LTF levels remain a controversial candidate biomarker for AD. To further investigate the potential role of LTF as a biomarker for AD, we examined LTF expression in both systemic and central compartments in the context of the AD diagnostic continuum in the largest dataset reported to date (N= 1,367). Our rationale was that if salivary LTF levels are directly associated with AD, similar associations might be detectable in other biological fluids – particularly in CSF, which is in closer contact with the neuropathological changes characteristic of the disease.

Our study was inspired by previous work suggesting a potential link between LTF and AD. Specifically, a 2017 study conducted a cross-sectional analysis using saliva samples and proposed salivary LTF as a promising biomarker to differentiate MCI and AD from healthy controls [27]. Two subsequent studies by the same group further supported this finding: one demonstrating that reduced salivary LFT could distinguish AD from other forms of dementia [28], and the other showed that LTF abundance is closely related to AD pathology [29]. Additional support came from a pilot study that reported significant associations between salivary LTF levels and cognitive performance, as measured by the Digit Span Memory Test and Mental Rotation Test scores [36]. Collectively, these studies suggest that reduced LTF levels in saliva may serve as a non-invasive biomarker for AD diagnosis. However, a study from an independent laboratory failed to replicate these findings in a consecutive, mixed memory clinic cohort [30], casting doubt on the reliability of salivary LTF as an AD biomarker. Each of these investigations were based on relatively small cohorts (N= 17–274)[27–30, 36], which may reduce statistical power and contribute to inconsistent findings [37]. Thus, it remains to be determined whether changes in LTF levels are part of the causal chain driving neurodegeneration, or merely epiphenomena reflecting systemic or local inflammation.

Additionally, the function of LTF is complex – it can exhibit both pro-inflammatory and anti-inflammatory properties depending on the context of response [31]. Despite growing interest in LTF as a potential biomarker or mediator in AD, several limitations complicate its study across different biological compartments. Beyond its role in inflammation, LTF can act as a mediator of both innate and adaptive responses by promoting the maturation, differentiation and activation of T-/ B-lymphocytes [38]. Its molecular complexity further challenges interpretation: LTF exists in multiple isoforms α, β, γ, and δ-LTF. The canonical isoform is primarily expressed in neutrophils and epithelial cell, secreted into exocrine inflamed tissue and fluid, while δ-LTF localizes to cytoplasm and act as a transcription factor [39–40]. Additionally, and importantly, iron-binding status further complicates LTF biology. Endogenous LTF exists in different iron-binding states including the iron-free (apo-LTF), native and iron-saturated forms (holo-LTF), which may have distinct biological functions and activities [41–42]. Apo-LTF mainly sequesters iron, while holo-LTF can activate immune signaling pathways via receptors such as LRP1 and CXCR4, influencing MAPK, AKT, and NF-κB pathways [43–44]. Proteolytic cleavage of LTF can also generate biologically active antimicrobial peptides known as lactoferricins, which exhibit different functional properties compared to native LTF [45]. Despite this molecular complexity, most current studies, including those evaluating its potential as an AD biomarker, use ELISA kits based on a single LTF antibody, which typically quantifies total or a single protein isoform, without distinguishing among various functional forms. Moreover, these studies often rely on correlation analyses between LTF levels and disease biomarkers, without addressing the biological significance or mechanistic relevance of inter-individual variation in LTF expression.

In our study, we aimed to explore the relationship between LTF levels in CSF and plasma with AD status, disease progression and AT(N) biomarkers. Unlike previous work focusing on salivary LTF, we used data derived from high-throughput SOMAscan proteomics on larger, well-characterized cognitive and biomarker cohorts. Our findings suggest that the relationship between LTF abundance and AD biomarkers is complex and compartment specific.

In our hands, LTF expression in CSF and Plasma shows a complex and inconsistent association with AD biomarkers. Three key observations highlight the complexity of interpreting LTF measurements using SOMAmer technology: 1. Lack of consistency between SOMAmers: The two SOMAmers targeting LTF (LTF1 and LTF2) showed markedly different distributions and negligible correlation in both CSF and plasma (Fig 1D). 2. Weak systemic-central correlation: Correlation between LTF SOMAmer signals between CSF and plasma was weak (Fig 1E). 3. Inconsistent association with AD biomarkers: In CSF, LTF1 displayed weak and even opposing correlations with the AD biomarker p-tau depending on LTF-high vs. low subgroups (Fig 1F, G) which may be caused by the variability in CSF protein abundance. Meanwhile, LTF1 is correlated with Qalb, but not for LTF2, suggesting that LTF1 may be more sensitive to barrier/inflammation-related signals, whereas LTF2 likely captures a different conformational or compartment-specific facet of LTF. Notably, CSF LTF2 levels were significantly higher in A+ than A-T-(N)- individuals (Fig.S3, adjusted p-value = 0.0102), and this difference was replicated in comparisons between A+T+ and A-T- groups (adjusted p-value = 0.0102). This pattern was not observed for LTF1 high group. Together, these findings indicate that LTF measurements obtained with SOMAmer reagents are heterogeneous across targets and fluids.

Importantly, our findings were further reinforced in the GNPC harmonized multi-cohort resource [34], where LTF1 and LTF2 again showed weak coupling, CSF–plasma concordance was essentially absent, and the most reproducible signal emerged at the network level, with LTF2 defining a cross-cohort plasma structure enriched for innate immune and granule biology (Supplementary Figures 4–7 and Supplementary Tables 7–13). Together, these results argue that LTF-related signals are assay-specific and compartment-specific, and that merging LTF1 and LTF2 would mask biologically meaningful differences.

An additional strength of the revised analysis is that it allows the main study cohort itself to be used as the biological anchor. When ACE/ContQ is treated as the reference dataset, the strongest reproducible signal again emerges for the plasma LTF2-centered network, whose shared recovery across independent GNPC cohorts argues against cohort-specific idiosyncrasy. Moreover, the corresponding positive consensus set forms a highly interconnected STRING network with a PPI enrichment far beyond chance expectation, reinforcing that this signal reflects coherent biology rather than recurrent statistical overlap. In this sense, the value of GNPC is not only that it supports structural replication, but that it helps reveal the cross-study biological program in which the main discovery signal operates.

This conclusion is further supported by published orthogonal evidence (Supplementary Figure 8): in an independent dual-platform CSF study, the LTF2 SomaScan signal showed strong agreement with mass spectrometry (r = 0.70, p = 6.8 × 10^−45^)[33], indicating that, at least, LTF2 is a bona fide LTF measurement rather than a platform artifact. The lack of association between LTF2 and AD phenotypes therefore cannot be readily dismissed as technical failure and instead suggests that prior LTF findings may reflect biology other than a direct surrogate of CNS LTF activity or AD biomarker status. We emphasize, however, that these data support measurement validity and structural interpretation, not standalone diagnostic or prognostic utility.

The most prominent studies evaluating LTF as an AD biomarker were conducted using saliva samples. Carro, et al. [27] first reported positive associations between salivary LTF and CSF Aβ42, and negative association with t-tau, suggesting that lower levels of salivary LTF are associated with increased markers of AD pathology. Later studies from the same group supported the discriminatory power of salivary LTF between AD and other dementia types [28], and also linked it to cortical Aβ load and brain integrity in aging adults [29]. A separate pilot study [36] found lower salivary LTF in participants with much more severe cognitive impairment. In contrast, our data from CSF and plasma cohorts challenge these findings, but do not necessarily negate them, as we were unable to replicate their work in saliva. We found there is trend of higher CSF LTF2 levels in MCI dementia compared with control (Kruskal-Wallis test; adjusted p-value = 0.05). These findings were consistent with those of another independent study [30], which analyzed 222 matched saliva and CSF samples from a consecutive, mixed memory clinic population and found both CSF and saliva LTF expression have higher LTF expression in MCI and AD participants than healthy control although their reported differences are not statically significant. This discrepancy may arise from difference in sample origin, population heterogeneity, or the biological nature of LTF secretion in different compartments. It is possible that LTF expression in CSF does not reflect the association previously described for salivary LTF in AD. If the reported salivary LTF findings are genuine, it is most likely capturing a biological signal that is not adequately measured by SOMAscan assays in CSF or plasma. Moreover, the lack of saliva samples in our study limits direct comparability.

Methodological work has emphasized the need to standardize salivary lactoferrin measurement procedures to achieve robust performance across settings [46], and mechanistic discussion has highlighted potential brain–immunity pathways that could couple cholinergic dysfunction and salivary gland innate responses [47]. In this context, our findings argue for caution in interpreting “lactoferrin” as a single interchangeable biomarker signal across fluids and platforms.

Another aspect is the technical considerations in SOMAmer-based measurements. According to SomaLogic documentation, LTF1 targets amino acids 20 – 710, while LTF2 spans the full LTF sequence. These reagents may bind distinct epitopes, requiring different dilutions and potentially detecting different isoforms or modifications of LTF. Consistent with this notion, LTF1 and LTF2 show distinct associations with Qalb, suggesting that structural and conformational differences may underlie compartment-specific behavior and, consequently, their differential relationships with disease biomarkers. This distinction may partly explain the divergent results we observed.

Our study benefits from two notable key methodological strengths, multi-tissue protein profiling, enabling cross-compartment comparison; and large-scale proteomic-level analysis, providing consistently measured insights into disease relevant proteins.

However, several limitations should be acknowledged. First, statistical power was limited in some subgroups, particularly healthy controls and the LTF1-high expressor group. Second, although paired CSF and plasma proteomics were available, the absence of saliva data precluded direct comparison with prior salivary LTF studies. Residual confounding from unmeasured factors such as oral health, diet, or medication use also cannot be excluded. In addition, no isoform-level validation currently exists for these SOMAmer measurements, and orthogonal data capable of clarifying which LTF form, proteoform, or molecular context being captured remain limited. Finally, our use of a heterogeneous memory clinic population likely diluted effects that might otherwise have been more readily detectable in a more phenotypically homogeneous setting.

These limitations do not, however, make the findings contradictory. Orthogonal mass spectrometry support indicates that LTF2 is a genuine LTF readout rather than a platform artifact, but a technically valid measurement need not be disease-relevant. Accordingly, the absence of robust associations between LTF2 and AD phenotypes is unlikely to reflect assay failure and instead suggests that prior LTF-related findings may be context-dependent, rather than evidence that this signal acts as a direct surrogate of CNS LTF activity or AD biomarker status. More broadly, although LTF shows some association with AD-related markers, particularly Aβ, its biomarker value remains uncertain because its behavior is shaped by molecular heterogeneity, compartment specificity, and assay context. The harmonized GNPC V1 resource does not yet provide the phenotypic depth required to fully resolve these issues, but it does provide a complementary strength at the network level: the plasma co-regulation meta-analysis centered on LTF2 recovered a robust cross-cohort functional structure enriched for innate immune and granule biology. Together with growing multi-omics evidence that protein abundance is often decoupled from genetic risk, these findings argue that regulatory and post-translational mechanisms may be more informative than bulk abundance alone. Future progress will therefore depend on assays that can resolve LTF isoforms, fragments, and iron-binding states with sufficient specificity to disentangle systemic from CNS-related biology and to identify the patient subsets in which LTF is mechanistically informative. The key question is no longer simply whether LTF is altered in AD, but which molecular form, in which compartment, and within which regulatory network carries biological and potentially therapeutic relevance.

## Methods

### Study cohort

We used the ACE Memory Clinic in Barcelona (Spain) cohort of well characterized CSF and matched plasma samples for all analysis in this study. Participants who presented for cognitive evaluation and met criteria for lumbar puncture were recruited. On the same day, both biological samples were collected, along with clinical, cognitive, and imaging data. Follow-up assessments were conducted annually. The cohort consisted of individuals with MCI, dementia, or subjective cognitive concerns. Diagnoses were made by a multidisciplinary team based on a comprehensive neuropsychological evaluation. Progression to dementia was monitored over time to classify participants as either converters or stable. Converters are defined as subjects who converted to dementia, including AD, vascular dementia, mixed dementia (AD with cerebrovascular disease), frontotemporal dementia, or dementia with Lewy bodies over the study period (from the baseline assessment to last available visit; max = 7.57 years, mean = 2.81 years), and were classified as MCI converters according to previous definitions [48]. All converter subjects had a Clinical Dementia Rating (CDR) score of 1. In contrast, those subjects who remained stable at follow-ups were classified as non-MCI converters. Sample processing followed standardized procedures [49]. Briefly, plasma centrifuged from blood, as well as CSF were stored at −80 °C for later use. Core AD CSF biomarkers (Aβ40, Aβ42, t-tau, and p-tau181) were quantified using validated immunoassay platforms, with predefined thresholds used for amyloid (A), tau (T), and neurodegeneration (N) (AT(N)) classification according to the 2018 NIA-AA Research Framework [50]. For details on sample acquisition and collection of additional information, refer to previous publications [34, 51–53].

### Protein analysis in CSF and plasma using the SOMAscan panel

Paired CSF and plasma proteomic data from 1,369 ACE cohort participants were generated using the SOMAscan 7K Assay Kit in the context of the HARPONE project and the GNPC [34, 52–53]. Briefly, this platform measures approximately 7,000 proteins per sample. This assay uses 50 μL of either CSF or plasma and employs modified DNA aptamers (SOMAmers) to quantify specific protein levels. Initially, proteins captured by immobilized aptamers, facilitated by streptavidin beads, and tagged with fluorescent markers. After removal of unbound proteins, the streptavidin beads are released using ultraviolet light. The protein-aptamer complexes were re-captured by monomeric avidin. The detail of the assay is described elsewhere [54]. Data was reported in normalized relative fluorescence units (RFU) values using the adaptive normalization by maximum likelihood (ANML) approach, following previously described procedures [54]. For the LTF gene (UniProt ID: P02788), two SOMAmers - Seq.14755.4 and Seq.2780.35 - were designed on the SOMAscan platform based on the target coding information provided by SomaLogic. These two SOMAmers have different dilution factors: 20% for Seq.14755.4 and 0.005% for Seq.2780.35 (Supplement Table S1). For clarity, we henceforth refer to Seq.14755.4 as LTF1 and Seq.2780.35 as LTF2 throughout this manuscript.

### Statistical methods

#### a. Identification of the association between LTF expression and dementia diagnosis

Prior to statistical analysis, SOMAmer measurements were log-transformed and standardized using z-score normalization due to the long tail of the SOMAmer signal among participants. The normality of LTF1/2 distribution was evaluated with Shapiro.test function in R, FDR was used for multiple correction together. To examine the association between LTF levels and AD-related biomarkers, the Spearman test was applied to compare the distributions of LTF SOMAmers, given their non-normal distribution. For LTF1, the raw data showed a bimodal distribution with a long tail after even log2 transformation, and our preliminary analyses suggested group-wise differences. We therefore further categorized participants into high (≥ mean + SD; LTF1-high) and low ((< mean + SD; LTF1-low) groups. To allow comparison, we also present the results before stratification in Figure 1, Figure S1, and Figure S3 as sensitivity analyses. In contrast, LTF2 did not show a similar distributional pattern, and therefore only outliers were removed using the standard approach. Spearman correlation analysis was used to assess the relationships between LTF levels (in CSF and plasma), AD CSF biomarker expression, and AD polygenic risk score (PRS) in a previous genome-wide association study [55]. Because LTF1 and LTF2 represent distinct SOMAmer signals with different target relationships and association patterns, analyses based on these two measures were treated separately, False discovery rate (FDR) correction was therefore applied within each prespecified analysis family for LTF1 and LTF2 rather than jointly across both signals in all following analyses. To address potential inaccuracies in p-values arising from tied ranks in Spearman correlation tests, genotype dosages were transformed into categorical values (0, 1, or 2), followed by Fisher’s exact test using the Fisher.test function with simulate.p.value = TRUE and B = 10,000, FDR was used for multiple correction. All statistical analyses were performed using R (v4.3). To evaluate whether the confounding factor affects the association between LTF1 and AD biomarkers (Aβ42, p-tau181), linear models were fit with sex, age, age2, principal components and reference genes GAGE2A (seq.18268.5) OPCML (seq.15622.13) signal as covariates. To perform principal components analysis (PCA), we filtered out samples with more than 10% protein that have outlier abundance (absolute value mean ± 3 SD), the outlier values left were imputed using function imputPCA in R package missMDA (v: 1.20). PCA was calculated using function prcomp in R package stats (4.5.1).

#### b. Assessment of associations between clinical traits and LTF protein expression

Patients were categorized into case and control groups based on AT(N) biomarker classification status. Individuals with an A-T-(N)- profile were assigned to the control group, while those with A+ (regardless of T or (N) status) were classified as cases. For further comparison, we also compared A+ v.s. A-, A+T+ v.s. A-T-, A+T+ v.s. A-T- (include A-T-(N)-). Participants were classified into three diagnostic categories: cognitively normal controls (including subjective cognitive decline (SCD)), mild cognitive impairment (MCI), and dementia. Due to the non-linear relationship between LTF and CSF biomarkers, we categorized the samples into high and low protein expressors according to the signals of LTF1 (High: > mean + SD; Low: < mean + SD) for correlation analysis. For LTF2, the outliers were Winsorized (mean ± 3 SD). FDR correction was applied separately within each prespecified analysis family.

#### c. Identification of the LTF CNS and systemic co-regulatory gene networks

To compare the LTF-associated genes with known co-regulated expression profiles, gene clusters containing 237 brain-expressed genes and 154 bone marrow–expressed genes were retrieved from the Human Protein Atlas database [56]. LTF-associated proteins were identified using Spearman correlation analysis via the cor.test function in R. Genes with an absolute Spearman correlation coefficient greater than 0.1 and ranked among the top 250 proteins by correlation were selected for the positively (+) and negatively (−) correlated groups, respectively. Multiple testing correction was applied using the FDR method. Fisher’s exact test was used to assess enrichment by comparing the identified genes from the proteomic data with gene sets from the Human Protein Atlas database.

#### d. Cox proportional hazards models

We applied a series of Cox proportional hazards regression models to assess the association between LTF1/LTF2 and the risk of conversion to dementia. Three hierarchical models were constructed to evaluate the robustness of the associations under different levels of covariate adjustment: Model 1 (unadjusted model): LTF1 or LTF2 signal (LTF) was entered independently to estimate its association with dementia conversion. Model 2 (demographic-adjusted model): Each variable was included in a Cox model adjusted for age at lumbar puncture (Age_LP), sex, years of education (Education), and baseline Mini-Mental State Examination (MMSE) score. Model 2 was treated as the primary confounder-adjusted model, accounting for demographic and baseline cognitive factors. Model 3 (Fully adjusted model): In addition to the covariates in Model 2, we further adjusted for PRS, CSF phosphorylated tau (p-tau181) levels, and APOE genotype. Model 3 was included as a more stringent model to test whether the association between LTF and conversion risk was independent of established AD-related risk/biomarker factors, including APOE genotype, PRS, and CSF p-tau181. Statistical tests were performed between the LTF1-high and -low groups. For LTF2, statistical significance was assessed by comparing samples with abundance above versus below the median. Hazard ratios (HRs), 95% confidence intervals (CIs), and p-values were reported for each model. Survival time was defined as the interval from MCI to dementia conversion or last follow-up assessment.

#### e. GNPC validation analyses

We performed independent validation analyses using harmonized proteomic matrices and metadata from the GNPC and are described extensively elsewhere [34]. This cohort constitutes the largest dataset of SOMAscan proteomic profiles from individuals with neurodegenerative diseases (AD, FTD, Parkinson’s disease dementia, Parkinson’s disease and amyotrophic lateral sclerosis) and cognitively unimpaired controls, collected across study sites in the USA, UK, and Europe. CSF and/or plasma samples were obtained from each participant at a single time point, along with demographic and limited clinical information. All participants provided written informed consent, and each contributing study received approval from its respective institutional ethics committee.

Analyses were restricted to baseline samples (visit = 1) and true biological samples (sample_type = “Sample”). CSF and EDTA plasma were analyzed separately using cohort-specific matrices (CSF and EDTAPlasma). The two LTF-targeting SOMAmer signals were analyzed as distinct features (LTF1: Seq.14755.4; LTF2: Seq.2780.35), consistent with the premise that multiple binders to the same protein may not behave interchangeably across conditions [32–33].

For correlation-based analyses, SOMAmer signals were log-transformed and standardized (z-scored) within each cohort-by-fluid matrix prior to testing, to reduce skew and support comparability across datasets [32]. We computed cohort-level Spearman correlations (ρ) and associated p-values for: (i) LTF1 vs. LTF2 within each fluid, (ii) LTF1 and LTF2 vs. the first two major proteomic components provided in GNPC matrices (Comp.1 and Comp.2), and (iii) paired cross-compartment concordance (CSF vs. EDTA plasma) within individuals in cohorts where person-level matching was available.

To test reproducibility of LTF-centered proteomic structure, we constructed cohort-specific co-regulatory networks by computing Spearman correlations between each LTF signal (LTF1 or LTF2) and all other SOMAmer features within a given cohort-by-fluid matrix. For each cohort, we extracted the top 250 positively and top 250 negatively correlated SOMAmers as directed LTF-centered signatures. Directed consensus markers were defined as SOMAmers present in the corresponding top-250 list in ⩾80% of datasets for each fluid (thresholds: ⩾4/5 for CSF; ⩾16/20 for EDTA plasma). For consensus markers, cohort-level correlation estimates were summarized by a Fisher z meta-analytic framework (z = atanh(ρ), weighted by N − 3) and transformed back to pooled correlation (tanh of the pooled z). As sensitivity summaries of cross-dataset support, we additionally combined cohort-level p-values using Fisher’s and Stouffer’s methods.

Finally, to assess whether replicated consensus structure reflected coherent biology rather than recurrent statistical noise, we performed pathway enrichment analysis on the EDTA plasma LTF2 positive directed consensus set using STRING annotations with FDR control [57]. All GNPC cohort-level statistics, consensus-marker lists with protein annotations, and enrichment results are provided in the Supplementary material (see Supplementary Tables 7–13 and Supplementary Figures. 4–8).

We additionally performed an ACE-anchored cross-cohort overlap analysis within GNPC, using ContQ (the ACE cohort within the harmonized framework) as the reference dataset. This analysis was restricted to the plasma LTF2-centered network, which represented the strongest reproducible GNPC signal, and quantified overlap with independent cohorts using overlap size, Jaccard index, overlap coefficient, Fisher’s exact test, and false-discovery-rate correction. The plasma LTF2 positive consensus set was further evaluated in STRING to assess whether the replicated proteins formed a significantly interconnected interaction network.

#### f. Ethics and consent

All procedures involving human participants were conducted in accordance with the Declaration of Helsinki and applicable Spanish biomedical research and data-protection regulations. The collection of cerebrospinal fluid and plasma samples and associated clinical data from the ACE Alzheimer Center Barcelona/Fundació ACE cohort was approved by the Clinical Research Ethics Committee of Hospital Clínic de Barcelona, Barcelona, Spain (reference HCB/2014/0494). The proteomic data analysed in this study were generated in the context of the HARPONE project, whose protocols were approved by the Ethics Committee of Hospital Universitari de Bellvitge, Barcelona, Spain (reference PR067/21). In addition, the present lactoferrin-focused research project received a favorable opinion from the Comité de Ética de la Investigación con medicamentos (CEIm) del Hospital Universitari de Bellvitge, Barcelona, Spain (reference PR235/25; Acta 12/26). Written informed consent was obtained from all participants or their legally authorized representatives prior to inclusion in the study. All contributing GNPC studies received approval from their respective institutional review boards or ethics committees, and all participants provided written informed consent.

#### g. Use of AI-assisted technologies

Declaration of Generative AI and AI-assisted technologies in the writing process: During the preparation of this work, the authors used ChatGPT (OpenAI) to improve language, readability, and phrasing. After using this tool, the authors reviewed and edited the content as needed and take full responsibility for the content of the publication. Codex (OpenAI) and Copilot (Microsoft) were used to assist with code drafting and troubleshooting for parts of the R-based data analysis workflow of GNPC analyses. All code outputs were subsequently reviewed, revised as needed, and validated by the authors.

#### h. Data availability

The individual-level ACE Alzheimer Center Barcelona/Fundació ACE/HARPONE datasets generated and/or analysed during the current study are not publicly available because they contain sensitive clinical, biomarker, and proteomic data from participants with cognitive impairment and dementia. Public deposition of these data would not be consistent with the informed consent provided by participants and with applicable ethical, institutional, and data-protection requirements. Data are available from the corresponding author upon reasonable request, subject to approval by the relevant ethics and institutional authorities and with permission from Fundació ACE/Ace Alzheimer Center Barcelona. The harmonized GNPC data used for validation analyses are available through the Alzheimer’s Disease Data Initiative/AD Discovery Portal. Access is subject to review and approval of a data use request and compliance with the GNPC Data Use Agreement and Publication Policies.

## Supplementary Material

1

## Banner of the Global Neurodegeneration Proteomics Consortium (GNPC)

- Charles H Adler, Mayo Clinic Arizona, Scottsdale, Arizona, USA
- Alireza Atri, Banner Sun Health Research Institute, Sun City, Arizona, USA
- Thomas G Beach, Banner Sun Health Research Institute, Sun City, Arizona, USA
- Graham Bearden, Alzheimer's Disease Data Initiative, Kirkland, WA
- James D. Berry, Sean M. Healey and AMG Center for ALS, Neurology
- Merce Boada, Ace Alzheimer Center Barcelona, Universitat Internacional de Catalunya, 08029 Barcelona, Spain; Biomedical Research Networking Centre in Neurodegenerative Diseases (CIBERNED), National Institute of Health Carlos III, 28029 Madrid, Spain
- Merle Bode, Hertie Institute for Clinical Brain Research, Neurodegenerative Diseases, Tübingen; German Center of Neurodegenerative Diseases, Department of Neurodegenerative Diseases, Tübingen 993
- Bradley Boeve, Mayo Clinic, Neurology Department, Rochester, MN
- Veronica Bot, Stanford University, The Phil and Penny Knight Initiative for Brain Resilience, Stanford, CA, USA; Stanford University, Wu Tsai Neurosciences Institute, Stanford, CA, USA; Stanford University, Graduate Program in Biomedical Engineering, Stanford, CA, USA
- Hillary Bounds, Gates Ventures, Seattle, WA
- Alfredo Cabrera-Socorro, Johnson & Johnson, NS TA, Beerse, Belgium
- Amanda Fernandez Cano, Ace Alzheimer Center Barcelona, Universitat Internacional de Catalunya, 08029 Barcelona, Spain; Biomedical Research Networking Centre in Neurodegenerative Diseases (CIBERNED), National Institute of Health Carlos III, 28029 Madrid, Spain
- Kaitlin B. Casaletto, University of California, San Francisco, Neurology Department, San Francisco, CA
- Richard J Caselli, Mayo Clinic Arizona, Scottsdale, Arizona, USA
- Yike Chen, Washington University School of Medicine, Department of Psychiatry, St. Louis, 63110, MO, USA.; NeuroGenomics and Informatics Center, Washington University School of Medicine, St. Louis, 63110, MO, USA.
- Matthew H.S. Clement, Alzheimer's Disease Data Initiative, Kirkland, WA
- Eric B. Dammer, Emory University School of Medicine, Atlanta, GA, USA; Emory University School of Medicine, Department of Biochemistry, Atlanta, GA, USA
- Sterre de Boer, Alzheimer Center Amsterdam, Neurology, Amsterdam UMC, Amsterdam, the Netherlands; Amsterdam Neuroscience, Amsterdam, the Netherlands
- Niels De Meirleir, Johnson & Johnson, NS TA, Beerse, Belgium
- Marta del Campo Milan, Barcelonaβeta Brain Research Center (BBRC), Pasqual Maragall Foundation, Barcelona, Spain; Hospital del Mar Research Institute, Barcelona, Spain
- Daisy Ding, Stanford University, The Phil and Penny Knight Initiative for Brain Resilience, Stanford, CA, USA; Stanford University, Wu Tsai Neurosciences Institute, Stanford, CA, USA; Stanford University, Graduate Program in Biomedical Engineering, Stanford, CA, USA
- Duc Duong, Emory University School of Medicine, Atlanta, GA, USA; Emory University School of Medicine, Department of Biochemistry, Atlanta, GA, USA 1022
- Maria Victoria Fernandez, Ace Alzheimer Center Barcelona, Universitat Internacional de Catalunya, 08029 Barcelona, Spain
- Lawrence Fourgeaud, Johnson & Johnson, NS TA, La Jolla, USA
- Raquel Puerta Fuentes, Ace Alzheimer Center Barcelona, Universitat Internacional de 1026 Catalunya, 08029 Barcelona, Spain; PhD Program in Biotecnology, Faculty of Pharmacy and Food Sciences, University of Barcelona, 08028 Barcelona, Spain
- Jordan Fuller, Gates Ventures, Seattle, WA
- Su Gao, Indiana Alzheimer's Disease Research Center, Indianapolis, IN; Indiana University School of Medicine, Department of Biostatistics & Health Data Science, Indianapolis, IN
- John Gibbons, Rush Alzheimer's Disease Center, Department of Neurological Sciences, Chicago, IL, USA
- Pablo Garcia Gonzalez, Ace Alzheimer Center Barcelona, Universitat Internacional de Catalunya, 08029 Barcelona, Spain; Biomedical Research Networking Centre in Neurodegenerative Diseases (CIBERNED), National Institute of Health Carlos III, 28029 Madrid, Spain
- Gyujin Heo, Washington University School of Medicine, Department of Psychiatry, St. Louis, 63110, MO, USA.; NeuroGenomics and Informatics Center, Washington University School of Medicine, St. Louis, 63110, MO, USA.
- Hilary Heuer, University of California, San Francisco, Neurology Department, San Francisco, CA
- Liping Hou, Johnson & Johnson, Spring House,
- Yen-Ning Huang, Indiana Alzheimer’s Disease Research Center, Indianapolis, IN; Indiana University School of Medicine, Department of Radiology & Imaging Sciences, Indianapolis, IN
- Alina Isakova, Stanford University, The Phil and Penny Knight Initiative for Brain Resilience, Stanford, CA, USA
- Clifford R. Jack, Jr, Mayo Clinic, Radiology
- Emily Kogan, Johnson & Johnson, JRD DSDH, Cambridge, USA
- Jessica B Langbaum, Banner Alzheimer's Institute, Phoenix, Arizona, USA
- Argentina Lario-Lago, University of California, San Francisco, Neurology Department, San Francisco, CA
- Shuwei Li, Johnson & Johnson, Spring House, USA
- Shiwei Liu, Indiana Alzheimer's Disease Research Center, Indianapolis, IN; Indiana University School of Medicine, Department of Radiology & Imaging Sciences, Indianapolis, IN
- Menghan Liu, Washington University School of Medicine, Department of Psychiatry, St. Louis, 63110, MO, USA.; NeuroGenomics and Informatics Center, Washington University School of Medicine, St. Louis, 63110, MO, USA.
- Marta Marquie, Ace Alzheimer Center Barcelona, Universitat Internacional de Catalunya, 08029 Barcelona, Spain; Biomedical Research Networking Centre in Neurodegenerative Diseases (CIBERNED), National Institute of Health Carlos III, 28029 Madrid, Spain
- Caitlin P. McHugh, Alzheimer's Disease Data Initiative, Kirkland, WA
- Martine Meyer, Johnson & Johnson, NS TA
- Silke Miller, Johnson & Johnson, NS TA, La Jolla, USA
- Elizabeth Mlynarski, Johnson & Johnson, JRD DSDH, Spring House, USA
- Diederik Moechars, Johnson & Johnson, NS TA, Beerse, Belgium
- Patricia Moran-Losada, Stanford University, The Phil and Penny Knight Initiative for Brain Resilience, Stanford, CA, USA; Stanford University, Wu Tsai Neurosciences Institute, Stanford, CA, USA; Stanford University School of Medicine, Department of Neurology and Neurological Sciences, Stanford, CA, USA
- Paige Opsahl, Gates Ventures, Seattle, WA
- Tamina Park, Indiana Alzheimer's Disease Research Center, Indianapolis, IN; Indiana University School of Medicine, Department of Radiology & Imaging Sciences, Indianapolis, IN
- Mukta Phatak, Alzheimer's Disease Data Initiative, Kirkland, WA
- Joni Lindbohm, MD, PhD, University College London, UCL Brain Sciences, London, UK; University of Helsinki, Clinicum, Helsinki, Finland
- Joseph Pick, Johnson & Johnson, Spring House, USA
- Yolande AL Pijnenburg, Alzheimer Center Amsterdam, Neurology Department, Amsterdam, the Netherlands; Amsterdam Neuroscience, Amsterdam, the Netherlands
- Michael Price, Michael J. Fox Foundation, New York, NY, USA
- Shannon Risacher, Indiana Alzheimer's Disease Research Center, Indianapolis, IN; Indiana University School of Medicine, Department of Radiology & Imaging Sciences, Indianapolis, IN
- Julio C. Rojas, University of California, San Francisco, Neurology Department, San Francisco, CA
- Howard J. Rosen, University of California, San Francisco, Neurology Department, San Francisco, CA
- Tamsin Sargood, Johnson & Johnson, Global Development, UK
- Claudia Schulte, Hertie Institute for Clinical Brain Research, Neurodegenerative Diseases, Tübingen; German Center of Neurodegenerative Diseases, Department of Neurodegenerative Diseases, Tübingen
- Weiwei Schultz, Johnson & Johnson, JRD DSDH, Titusville, USA
- Geidy E Serrano, Banner Sun Health Research Institute, Sun City, Arizona, USA
- Nicholas T. Seyfried, Emory University School of Medicine, Atlanta, GA, USA; Emory University School of Medicine, Department of Neurology, Atlanta, GA, USA; Emory University School of Medicine, Department of Biochemistry, Atlanta, GA, USA
- Todd Sherer, Michael J. Fox Foundation, New York, NY, USA
- Emily Smith, Indiana Alzheimer's Disease Research Center, Indianapolis, IN; Indiana University School of Medicine, Department of Radiology & Imaging Sciences, Indianapolis, IN
- Adam M. Staffaroni, University of California, San Francisco, Neurology Department, San Francisco, CA
- Russell H. Swerdlow, University of Kansas Alzheimer's Disease Research Center, Kansas City, Kansas, USA; University of Kansas, Neurology, Kansas City, Kansas, USA
- Shinya Tasaki, Rush Alzheimer's Disease Center, Department of Neurological Sciences, Chicago, IL, USA
- Charlotte Teunissen, Neurochemistry Laboratory, Neurology Department, Amsterdam, the Netherlands; Amsterdam Neuroscience, Amsterdam, the Netherlands
- Terri G. Thompson, OnPoint Scientific, Inc, San Diego, CA, USA
- Qu Tian, NIH/NIA
- Jigyasha Timsina, Washington University School of Medicine, Department of Psychiatry, St. Louis, 63110, MO, USA.; NeuroGenomics and Informatics Center, Washington University School of Medicine, St. Louis, 63110, MO, USA.
- Abolfazl Doostparast torshizi, Johnson & Johnson, Spring House, USA
- Sergi Valero, Ace Alzheimer Center Barcelona, Universitat Internacional de Catalunya, 08029 Barcelona, Spain; Biomedical Research Networking Centre in Neurodegenerative Diseases (CIBERNED), National Institute of Health Carlos III, 28029 Madrid, Spain
- Wiesje M van der Flier, Alzheimer Center Amsterdam, Neurology Department, Amsterdam, the Netherlands; Amsterdam Neuroscience, Amsterdam, the Netherlands; Epidemiology 1122 and Data Science, Amsterdam UMC
- Julia D. Webb, University of California, San Francisco, Neurology Department, San 1124 Francisco, CA
- Bryan K Woodruff, Mayo Clinic Arizona, Scottsdale, Arizona, USA
- Ying Xu, Washington University School of Medicine, Department of Psychiatry, St. Louis, 63110, MO, USA.; NeuroGenomics and Informatics Center, Washington University School of Medicine, St. Louis, 63110, MO, USA.
- Mariet A. Younkin, Mayo Clinic, Neurology, Rochester, MN
