# Supplementary material for: Lactoferrin Levels in Cerebrospinal Fluid Exhibit Differential Associations with Alzheimer’s Disease
Source: Res Sq. 2026 Jul 7:rs.3.rs-9678397. Preprint. [Version 1] doi: 10.21203/rs.3.rs-9678397/v1 (PMC13370628; doi:10.21203/rs.3.rs-9678397/v1)
Supplement: 1 [file NIHPPRS9678397V1-supplement-1.pdf]

1. Supplementary Figures

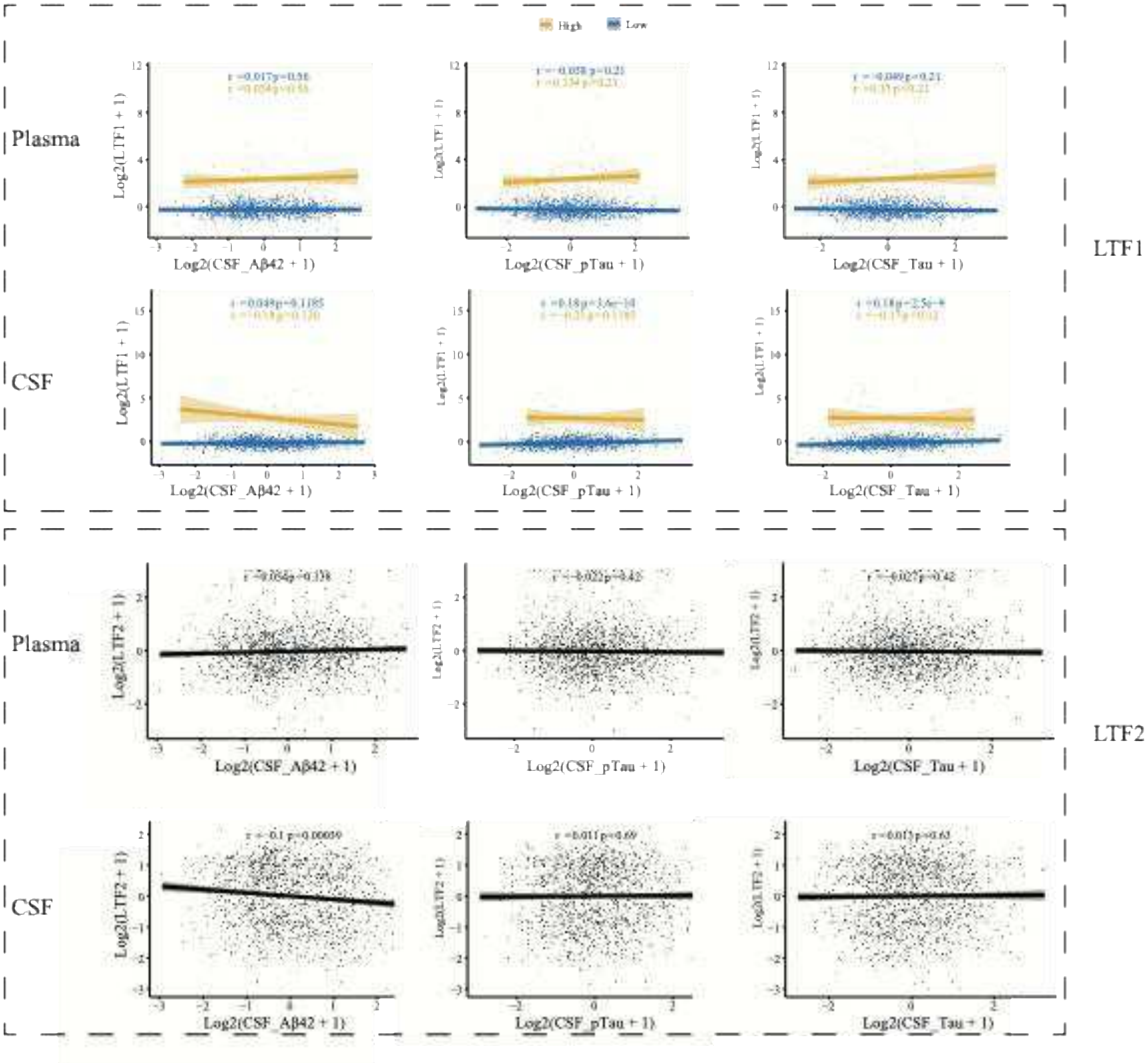

Fig. S1. Scatter plots showing the correlations between lactoferrin (LTF) 1/2 and Alzheimer's disease (AD) biomarkers (Aβ42, p-tau, and t-tau). The top row shows correlations with LTF1, and the bottom row with LTF2. LTF1 is stratified by LTF1 expression—LTF1-high (yellow; n= 123) vs LTF1-low (blue; n= 1,244). For LTF2, correlations were computed across all samples (n= 1,367). Spearman correlation coefficients (r) and corresponding p-values (*p*) are indicated in each panel. False Discovery Rate (FDR) was used to perform multiple corrections for LTF1/LTF2 separately.

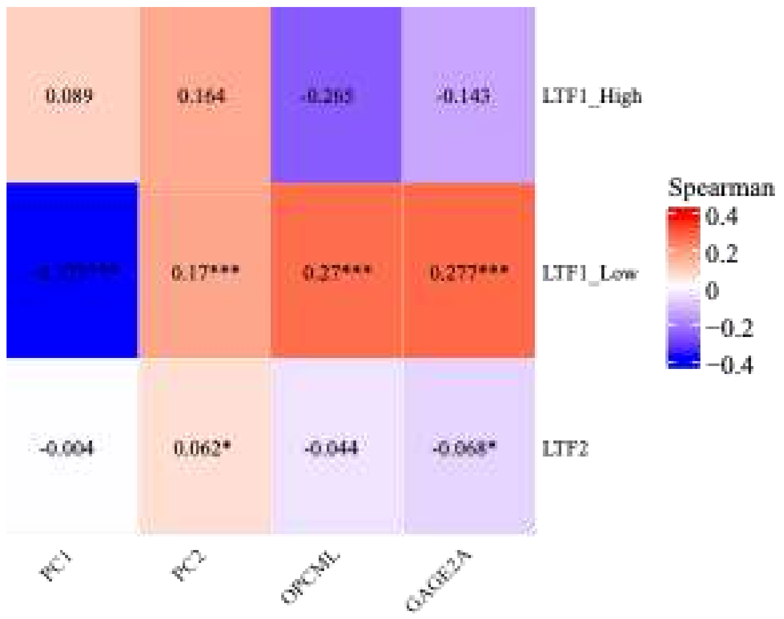

Fig. S2. **Spearman correlation heatmap between cerebrospinal (CSF) lactoferrin (LFT) 1/2, the first two proteomic principal components (PC1–PC2) and reference genes (OPCML, GAGE2A).** Cells display Spearman's ρ; color encodes effect size. Significance: *, $p < 0.05$; **, $p < 0.01$; **, $p < 0.001$.

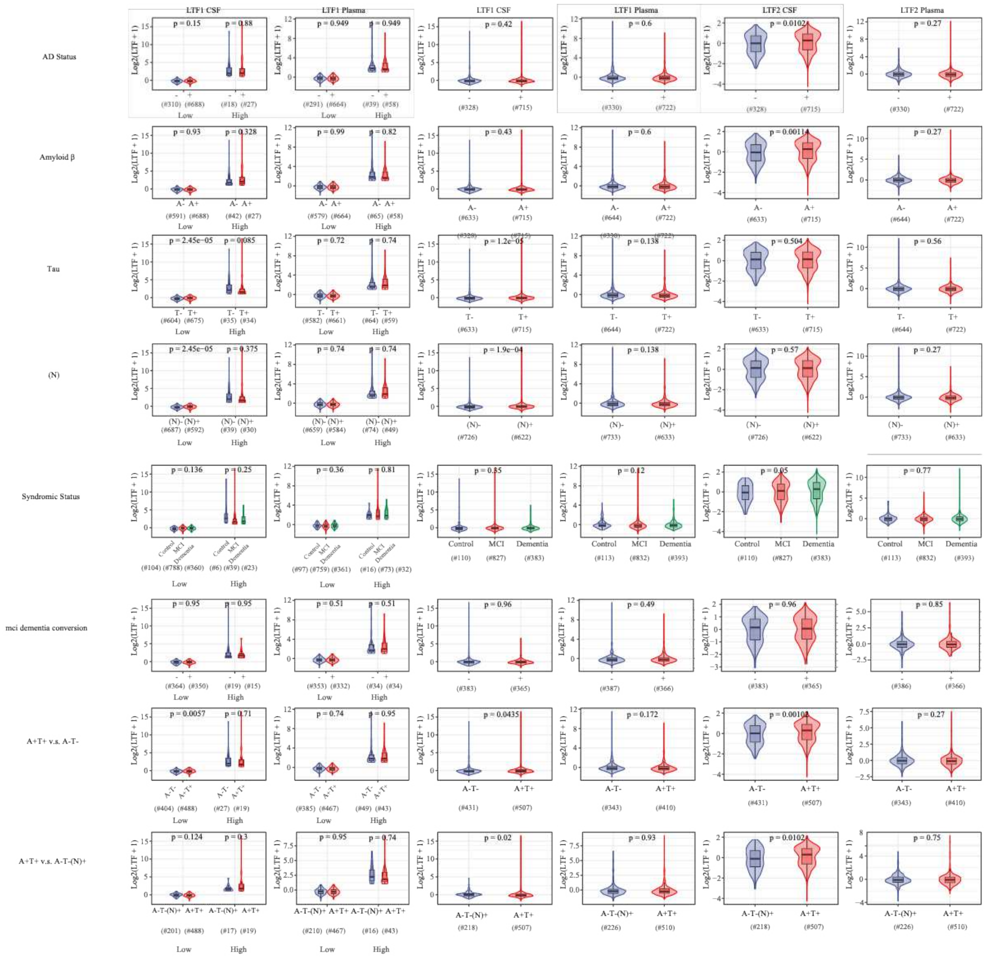

Fig. S3. Comparison of lactoferrin (LTF) 1 and 2 levels across AD biomarker profiles, clinical diagnosis, APOE genotypes, and longitudinal conversion status.

Each row represents a comparison between groups stratified by APOE genotype, dementia diagnosis, A/T/N biomarker positivity, or longitudinal conversion status. Columns 1 – 2 show CSF and plasma LTF1 levels stratified by LTF1 abundance (high, low) respectively; columns 3 – 4 show the CSF and plasma LTF1 levels before stratification. Columns 5 – 6 show CSF and plasma LTF2 levels. In each plot, different colors were used to discriminate the status of diagnosis, AD biomarkers, APOE genotypes or conversion status respectively. Statistical comparisons were performed using the Kruskal-Wallis test. False discovery rate (FDR) correction was therefore applied within each prespecified analysis family for LTF1 and LTF2 separately.

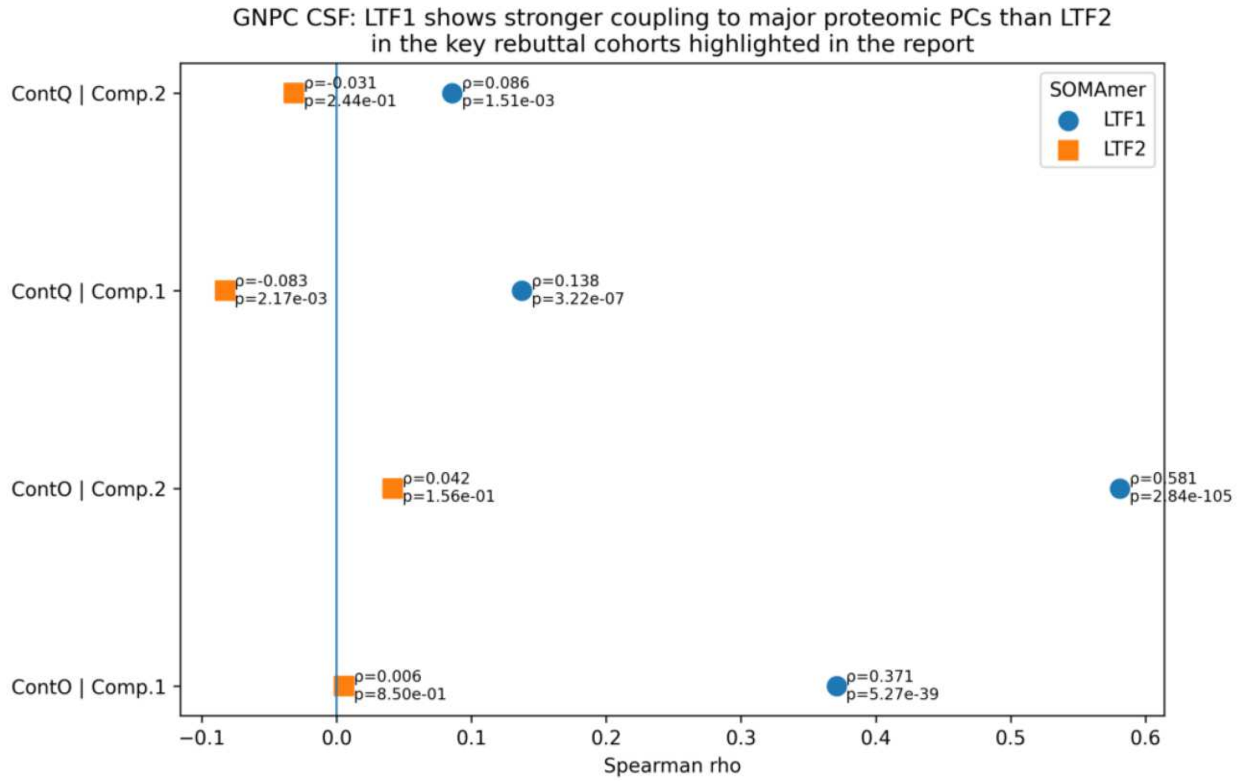

Fig. S4. Global Neurodegeneration Proteomics Consortium (GNPC) cerebrospinal fluid (CSF) replication of differential coupling of lactoferrin (LTF) 1 vs. LTF2 to proteomic components.

Legend: Cohort-level Spearman correlations of CSF LTF1 and LTF2 with Comp.1 and Comp.2 across GNPC CSF datasets at baseline. Panels may show per-cohort point estimates. Full values in Table S7.

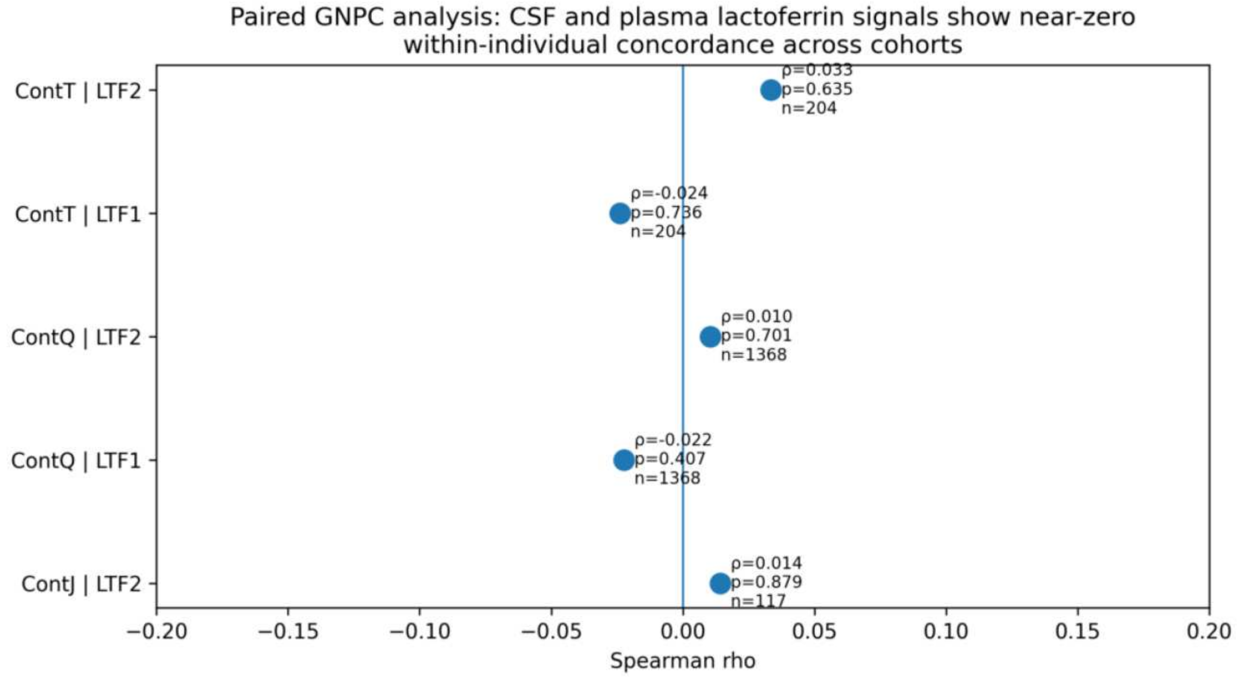

Fig. S5. Paired Global Neurodegeneration Proteomics Consortium (GNPC) cerebrospinal fluid (CSF)–plasma comparisons show near-zero within-individual concordance.

Legend: Within-individual concordance between CSF and EDTA plasma lactoferrin signals in cohorts with matched sampling, reported separately for LTF1 and LTF2. Full statistics in Table S9.

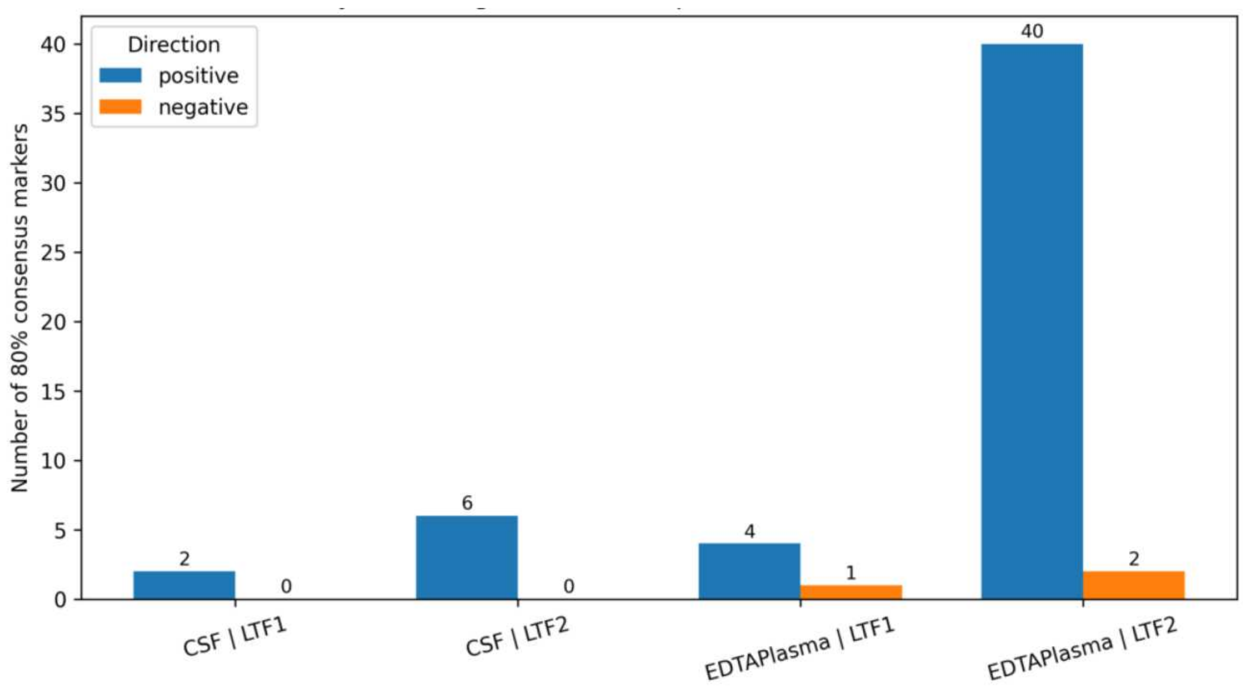

Fig. S6. Cross-cohort consensus network replication summary highlights stronger reproducibility for plasma lactoferrin (LTF) 2.

Legend: Number of directed 80% consensus markers for LTF1 vs. LTF2 by fluid and direction (positive/negative). Representative plasma LTF2 consensus proteins are annotated directly on the plot. Corresponding consensus lists are found in Table S10 and meta-analysis in Table S11.

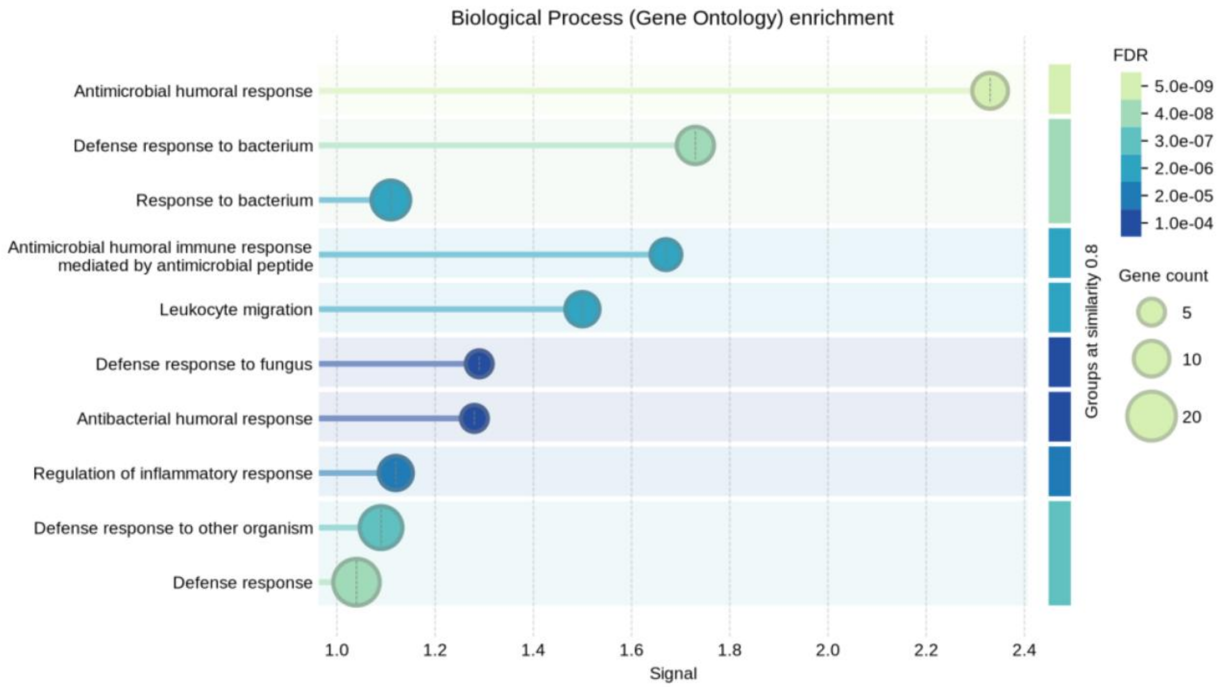

Fig. S7. Enrichment analysis of the plasma lactoferrin (LTF) 2 positive consensus network.

Legend: Dot plot or bar plot of top enriched terms for the EDTA plasma LTF2 positive directed consensus set, emphasizing innate immune, neutrophil, and secretory granule biology. Full enrichment results displayed in Table S13.

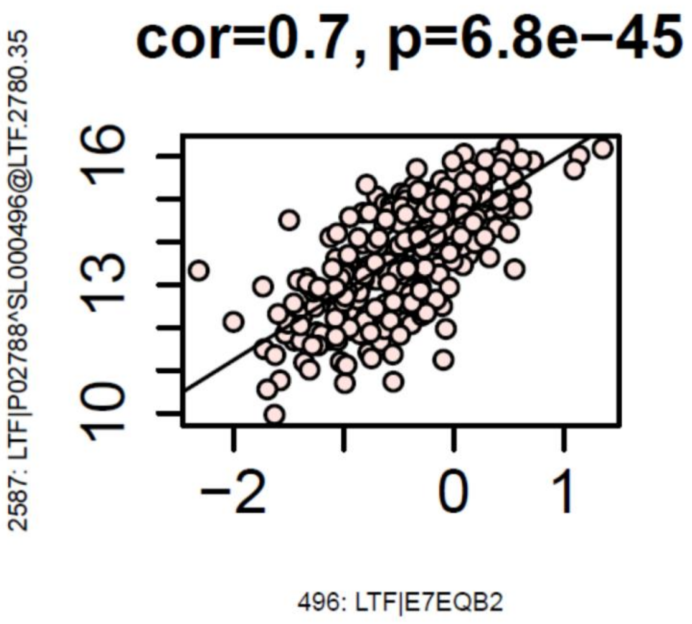

Fig. S8. Orthogonal agreement between the lactoferrin (LTF) 2 SomaScan signal and mass spectrometry measurement in cerebrospinal fluid (CSF). Correlation between the lactoferrin SOMAmer LTF2 (Seq.2780.35 / P02788) measured by SomaScan and the corresponding mass spectrometry signal in CSF, showing strong positive association. This data supports that LTF2 captures a reproducible signal also detected by an orthogonal proteomic platform (public information extracted from reference 33).

2. Supplementary Tables

Table S1. Annotation of the two SOMAmers that target to LTF**.**

Table S2. Demographic and clinical data of the participants in the ACE cohort.

Abbreviations: LP: lumbar puncture; MCI, mild cognitive impairment; AD, Alzheimer's disease dementia; MMSE, Mini Mental State Examination; LTF, Lactoferrin; NA, not applicable; SD, standard deviation.

Table S3. Results of the linear regression between cerebrospinal fluid (CSF) Aβ42, p-tau and LTF1 using sex, age, age^2, reference gene, PC1, PC2. Results are shown for models adjusting for each covariate individually and for a fully adjusted model including all covariates simultaneously. model1: Aβ42 ~ LTF1

model2: Aβ42 ~ LTF1 + poly(Age_LP, 2); model3 : Aβ42 ~ LTF1 + sex_1M_2F; model4 : Aβ42 ~ LTF1 + seq.18268.5_GAGE2A; model5 : Aβ42 ~ LTF1 + PC1; model6 : Aβ42 ~ LTF1 + PC2; model7 : Aβ42 ~ LTF1 + poly(Age_LP, 2) + sex_1M_2F + seq.18268.5_GAGE2A + PC1 + PC2; model8 : p-tau ~ LTF1; model9 : p-tau ~ LTF1 + poly(Age_LP, 2); model10 : p-tau ~ LTF1 + sex_1M_2F; model11 : p-tau ~ LTF1 + seq.18268.5_GAGE2A; model12 : p-tau ~ LTF1 + PC1; model13 : p-tau ~ LTF1 + PC2; model14 : p-tau ~ LTF1 + poly(Age_LP, 2) + sex_1M_2F + seq.18268.5_GAGE2A + PC1 + PC2.

Table S4. Results of the linear regression between cerebrospinal fluid (CSF) Aβ42, p-tau and LTF1 using sex, age, age^2, reference gene, PC1, PC2 as covariates in LTF1 high expressing population. Results are shown for models adjusting for each covariate individually and for a fully adjusted model including all covariates simultaneously. model1: Aβ42 ~ LTF1; model2 : Aβ42 ~ LTF1 + poly(Age_LP, 2); model3 : Aβ42 ~ LTF1 + sex_1M_2F; model4 : Aβ42 ~ LTF1 + seq.18268.5_GAGE2A; model5 : Aβ42 ~ LTF1 + PC1; model6 : csf_abeta_42 ~ LTF1 + PC2; model7 : Aβ42 ~ LTF1 + poly(Age_LP, 2) + sex_1M_2F + seq.18268.5_GAGE2A + PC1 + PC2; model8 : p-tau ~ LTF1; model9 : p-tau ~ LTF1 + poly(Age_LP, 2); model10 : p-tau ~ LTF1 + sex_1M_2F; model11 : p-tau ~ LTF1 + seq.18268.5_GAGE2A; model12 : p-tau ~ LTF1 + PC1; model13 : p-tau ~ LTF1 + PC2; model14 : p-tau ~ LTF1 + poly(Age_LP, 2) + sex_1M_2F + seq.18268.5_GAGE2A + PC1 + PC2.

Table S5. Results of the linear regression between cerebrospinal fluid (CSF) Aβ42, p-tau and LTF1 using sex, age, age^2, reference gene, PC1, and PC2 as covariates in LTF1 low expressing population. Results are shown for models adjusting for each covariate individually and for a fully adjusted model including all covariates simultaneously. model1: Aβ42 ~ LTF1; model2 : Aβ42 ~ LTF1 + poly(Age_LP, 2); model3 : Aβ42 ~ LTF1 + sex_1M_2F; model4 : Aβ42 ~ LTF1 + seq.18268.5_GAGE2A; model5 : Aβ42 ~ LTF1 + PC1; model6 : Aβ42 ~ LTF1 + PC2; model7 : Aβ42 ~ LTF1 + poly(Age_LP, 2) + sex_1M_2F + seq.18268.5_GAGE2A + PC1 + PC2; model8 : p-tau ~ LTF1; model9 : p-tau ~ LTF1 + poly(Age_LP, 2); model10 : p-tau ~ LTF1 + sex_1M_2F; model11 : p-tau ~ LTF1 + seq.18268.5_GAGE2A; model12 : p-tau ~ LTF1 + PC1; model13 : p-tau ~ LTF1 + PC2; model14 : p-tau ~ LTF1 + poly(Age_LP, 2) + sex_1M_2F + seq.18268.5_GAGE2A + PC1 + PC2.

Table S6. Results of the linear regression between cerebrospinal fluid (CSF) Aβ42, p-tau181 and LTF2 using sex, age, age^2, reference gene, PC1, PC2 as covariates. Results are shown for models adjusting for each covariate individually and for a fully adjusted model including all covariates simultaneously. model1 : Aβ42 ~ LTF2; model2 : Aβ42 ~ LTF2 + poly(Age_LP, 2); model3 : Aβ42 ~ LTF2 + sex_1M_2F; model4 : Aβ42 ~ LTF2 + seq.18268.5_GAGE2A; model5 : Aβ42 ~ LTF2 + PC1; model6 : Aβ42 ~ LTF2 + PC2; model7 : Aβ42 ~ LTF2 + poly(Age_LP, 2) + sex_1M_2F + seq.18268.5_GAGE2A + PC1 + PC2; model8 : p-tau ~ LTF2; model9 : p-tau ~ LTF2 + poly(Age_LP, 2); model10 : p-tau ~ LTF2 + sex_1M_2F; model11 : p-tau ~ LTF2 + seq.18268.5_GAGE2A; model12 : p-tau ~ LTF2 + PC1; model13 : p-tau ~ LTF2 + PC2; model14 : p-tau ~ LTF2 + poly(Age_LP, 2) + sex_1M_2F + seq.18268.5_GAGE2A + PC1 + PC2.

Table S7. Global Neurodegeneration Proteomics Consortium (GNPC) cohort-level Spearman correlations for LTF1 and LTF2 in cerebrospinal fluid (CSF).

Cohort-level Spearman correlations (ρ), p-values, and N for CSF datasets at baseline (visit = 1; sample_type = “Sample”). Includes LTF1 vs. LTF2 and correlations of LTF1 and LTF2 with GNPC-provided Comp.1 and Comp.2; age_at_visit and Clinical Demential Rating (CDR) score may be included where available. Non-estimable correlations should be indicated where variance was insufficient.

Table S8. Global Neurodegeneration Proteomics Consortium (GNPC) cohort-level Spearman correlations for LTF1 and LTF2 in baseline EDTA plasma. Includes LTF1 vs. LTF2 and correlations of LTF1/LTF2 with Comp.1 and Comp.2, with clinical covariates where available.

Table S9. Paired Global Neurodegeneration Proteomics Consortium (GNPC) cerebrospinal fluid (CSF)–EDTA plasma within-individual concordance for LTF1 and LTF2. Within-individual Spearman correlations between CSF and EDTA plasma for matched individuals in cohorts with paired sampling. Data reported separately for LTF1 and LTF2 with N, ρ, and p-values.

Table S10. Directed 80% cross-dataset consensus marker lists for LTF1 and LTF2 networks. Directed consensus markers defined by membership in top-250 positive or top-250 negative lactoferrin-centered correlation signatures in ≥80% of datasets within each fluid (CSF ≥4/5; EDTA plasma ≥16/20). Report marker identifiers, protein annotations, number/proportion of datasets, and direction.

Table S11. Meta-analysis of directed consensus markers across Global Neurodegeneration Proteomics Consortium (GNPC) datasets. For each directed consensus marker, cohort-specific Spearman correlations were summarized using Fisher z meta-analysis weighted by (n − 3), with pooled correlation, standard error, z-statistic, and two-sided p-value. Fisher’s and Stouffer’s combined p-values may be provided as sensitivity summaries.

Table S12. Overlap summary of consensus networks across fluids. Directed overlap statistics comparing cerebrospinal fluid (CSF) vs. EDTA plasma consensus sets, optionally including positive vs. negative comparisons within fluid. Reported overlap counts and Jaccard indices to highlight compartment specificity.

Table S13. Functional enrichment results for the EDTA plasma LTF2 positive directed consensus set using STRING annotation resources with false discovery rate (FDR) correction. Terms are reported with gene counts and adjusted p-values (FDR).

Supplementary Files

This is a list of supplementary files associated with this preprint. Click to download.

- SupplementTableswithGNPC.xlsx
